# Evaluating the use of HILIC in large-scale, multi dimensional proteomics: Horses for courses?

**DOI:** 10.1016/j.ijms.2015.07.029

**Published:** 2015-11-30

**Authors:** Dalila Bensaddek, Armel Nicolas, Angus I. Lamond

**Affiliations:** Centre for Gene Regulation and Expression, School of Life Sciences, University of Dundee, Dow Street, Dundee DD1 5EH, United Kingdom

**Keywords:** RP, reverse-phase, HILIC, hydrophilic interaction liquid chromatography, hSAX, hydrophilic strong anion exchange, PTM, post-translational modification, 2D-LC, HILIC, PTM, Multi-dimensional proteomics, Chromatography, Mass-spectrometry

## Abstract

Despite many recent advances in instrumentation, the sheer complexity of biological samples remains a major challenge in large-scale proteomics experiments, reflecting both the large number of protein isoforms and the wide dynamic range of their expression levels. However, while the dynamic range of expression levels for different components of the proteome is estimated to be ∼10^7–8^, the equivalent dynamic range of LC–MS is currently limited to ∼10^6^. Sample pre-fractionation has therefore become routinely used in large-scale proteomics to reduce sample complexity during MS analysis and thus alleviate the problem of ion suppression and undersampling. There is currently a wide range of chromatographic techniques that can be applied as a first dimension separation. Here, we systematically evaluated the use of hydrophilic interaction liquid chromatography (HILIC), in comparison with hSAX, as a first dimension for peptide fractionation in a bottom-up proteomics workflow. The data indicate that in addition to its role as a useful pre-enrichment method for PTM analysis, HILIC can provide a robust, orthogonal and high-resolution method for increasing the depth of proteome coverage in large-scale proteomics experiments. The data also indicate that the choice of using either HILIC, hSAX, or other methods, is best made taking into account the specific types of biological analyses being performed.

## Highlights

• HILIC provides a robust and reproducible off-line LC method for 2D-LC deep proteomics workflows.
• HILIC is part-orthogonal to hSAX fractionation.
• HILIC-based workflow identified ∼81,000 peptides from >9000 proteins with an average sequence coverage of ∼26%.
• HILIC is well suited for the study of hydrophilic PTMs.

## Graphical abstract

**Figure:**
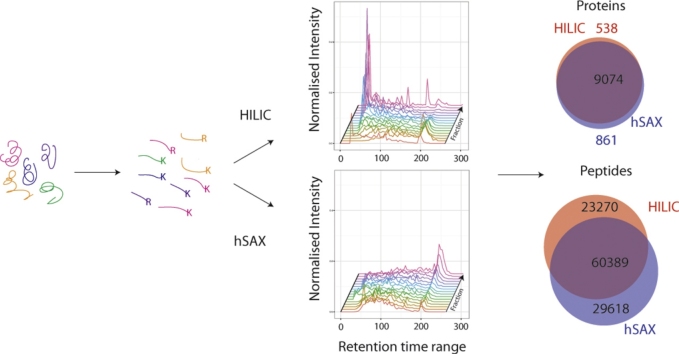

## 1 Introduction

It is not so long since researchers would have counted themselves lucky to identify a few tens of proteins from a single shotgun proteomics experiment. However spectacular progress has been made in improving the efficiency of protein detection at multiple levels, including experiment design and protocols, sample preparation workflows, LC–MS instrumentation, and in silico analysis. As a result, it is now possible to identify a large proportion of a steady state cell proteome [1] in a single experiment, either with or without, fractionation [2], [3]. Furthermore, it is also possible to describe additional proteome dimensions, such as protein turnover rate, cell cycle-specific changes, post-translational modifications and subcellular localization [4].

A limitation of early shotgun proteomics experiments is that the resulting data were predominantly one dimensional: whether the sample was derived from either a whole organism, tissue, cultured cells or a purified organelle or subcellular fraction, the final result was typically a list of identified protein groups with limited quantitative information. However, to describe a cell proteome in a way that is both accurate and with maximum physiological relevance for understanding biological mechanisms, it is important not only to include quantitation of protein expression levels, but also to resolve protein groups into single isoforms (i.e., addressing the so-called “isoform inference” problem associated with bottom-up proteomics), while also addressing such parameters as the subcellular distribution of proteins and the presence of post-translational modifications (PTMs). This could also be combined with analysis of additional proteome properties, for example higher order protein complexes, cell-cycle dependent variations of the proteome, and/or the rate of protein turnover. This combined analysis approach has been referred to as either “Next Generation Proteomics” or, perhaps more accurately, “multidimensional proteomics” [5]. A major advantage of the multidimensional characterization of cell proteomes is the ability to mine the resulting data to establish correlations between different properties, for example linking the subcellular location of a protein with either a specific isoform or post-translational modification [6], [7]. This can generate useful hypotheses regarding the functional significance of such correlations that can be evaluated directly in follow-on experiments.

The comprehensive description of the proteome has to overcome several analytical challenges, including the inherent complexity of protein types in cell extracts and the wide dynamic range of protein expression levels. Thus, taking into account isoforms and PTMs, a cell proteome can potentially comprise several hundred thousands of protein isotypes, spanning at least five or more orders of magnitude in abundance. As a result, a wide range of fractionation strategies for peptides and proteins have become an integral part of proteomics workflows, with the general aim of reducing the sample complexity to a manageable level prior to tandem mass spectrometry (MS/MS) analysis. This in turn reduces ion suppression effects and maximizes the number of peptides that are effectively transferred to the gas phase as gaseous ions, sequenced and successfully identified.

The most commonly used multidimensional LC setup involves two chromatographic separation steps, or dimensions, and is referred to as two-dimensional liquid chromatography (2D-LC). In theory, any type of chromatographic separation can be used at either the protein, or peptide level, including ion exchange chromatography (IEC), standard and high pH reversed phase (RP), hydrophobic interaction chromatography (HIC) and/or size exclusion chromatography (SEC). In bottom up proteomics, however, 2D-LC is commonly a combination of an off-line chromatographic method followed by RP-LC directly coupled to the mass spectrometer.

2D-LC has the potential to dramatically improve the separation power of chromatography, with its performance depending both on the peak capacity of the two chromatographic dimensions and their degree of orthogonality. In chromatography, the term ‘orthogonal’ is used to refer to a complementary method of fractionation from the initial fractionation, so that orthogonal chromatographic systems are typically based on the use of different physico-chemical properties to separate peptides. In this way, a more effective overall separation of the original peptide mixture is provided, ultimately allowing more peptides to be identified. A number of studies have previously investigated this concept of orthogonality in 2D-LC separation [8], [9], [10], [11]. For instance, it is common to use ion exchange chromatography prior to RP-LC, as the two techniques are complementary and compatible. In this case the peptides are separated by charge in one dimension and hydrophobicity in the second dimension.

In addition to ion exchange chromatography, other approaches also offer orthogonality with RP-LC, such as hydrophilic interaction liquid chromatography (HILIC) [12], which has recently emerged as a popular chromatographic mode for the separation of hydrophilic analytes. HILIC operates on the basis of hydrophilic interactions between the analytes and the hydrophilic stationary phase, with either highly polar, or hydrophilic compounds interacting most strongly [13].

There are several different HILIC stationary phases [14], [15], including derivatized silica material, which can be neutral, such as the cation exchanger polysulfoethyl A [12], the weak cation exchanger Polycat A [16], the weak anion exchanger PolyWAX [17], TSKgel amide-80 [18], [19] and zwitterionic ZIC-HILIC [20], [21]. While these supports differ in the exact chromatographic mechanism by which they separate analytes, they all generate a hydrophilic layer around the functional groups, which strongly interacts with either polar, or hydrophilic compounds. Therefore, HILIC can in practice be viewed as “reversed RP”.

Gradient elution in HILIC can be achieved by increasing the polarity of the mobile phase, either by reducing the concentration of organic solvent, or by increasing the salt concentration, depending on the stationary phase. When peptides are separated using a non-ionic stationary phase, such as TSKgel Amide-80, an inverse acetonitrile gradient is most convenient. If the separation is carried out using ionic packing, such as that contained in PolyHydroxyethyl A columns, an increasing salt gradient is normally used. When using the TSKgel Amide-80 stationary phase, it is necessary to include a pairing agent, such as TFA, in the mobile phase to prevent ionic interactions between peptide residues and residual silanol groups on the silica surface. The use of weaker acids was reported to negatively affect the chromatography by reducing peptide elution and broadening peaks [22]. In the absence of acid, the separation is based on mixed mode (polar interactions and ionic interactions) [22]. TFA results in ion suppression when the eluent is directly sprayed at the sampling region of the mass spectrometer, however it is not an issue at all to use in off-line preparative LC, as is the case of a 2D-LC set up. One of the major issues affecting the ability to combine HILIC and RPLC in an online setup has been the incompatibility of the solvents used in both dimensions. However, recently Di palma et al., reported a robust 2D-LC setup allowing the combination of the separation modes [23].

A number of previous studies have compared the performance of HILIC against other chromatographic separation modes, including strong cation exchange (SCX) and reversed phase (RP). SCX is commonly used for peptide fractionation in 2D-LC setups. However, SCX suffers from low resolution as well as the additional requirement for desalting, which may result in losses, especially in phosphorylated peptides and hydrophilic peptides in general. Studies that have compared ZIC-HILIC and SCX side by side have reported that the former has higher resolution and results in increased numbers of identifications [20], [24]. HILIC also performed better on iTRAQ-labelled samples [24], [25] and was reported to reduce iTRAQ ratio-compression, a fact which has been attributed to its higher resolution [26].

The efficacy of HILIC for the separation of polar compounds has been effectively exploited in the study of PTMs, including carbohydrates [27], [28], glycopeptides [29], [30], [31] and phosphopeptides [18], [32].

HILIC has also been used in combination with selective phosphopeptide enrichment methods, such as either immobilized metal affinity chromatography (IMAC) [33], [34], or TiO_2_ [35] enrichment in different orders. *Phospho-enrichment first*: For example, Albuquerque et al. [36] reported the development of a multidimensional chromatography method combining IMAC, HILIC and RP-LC to purify and fractionate phosphopeptides. They showed that HILIC was largely orthogonal to RP-HPLC for phosphopeptide enrichment. Wu et al. [37] combined dimethyl labelling, IMAC separation and HILIC fractionation to identify 2857 unique phosphorylation sites in the MCF7 breast cancer cell line. *HILIC first*: Annan and Mcnulty reported the use of HILIC as a pre-enrichment step prior to IMAC-based phospho-enrichment for large scale proteomics studies [18]. This approach was successfully adopted in other studies [38], [39].

HILIC has also been employed either before, or after, phosphoenrichment. Thus, Engholm-Keller et al. reported a combination of a large-scale phosphoproteomics protocol prior to HILIC fractionation and TiO_2_ enrichment [40]. By using sequential elution from IMAC (SIMAC) [41], mono-phosphorylated peptides are separated from multiphosphorylated peptides. Non-phosphorylated and monophosphorylated peptides were further fractionated using HILIC, followed by TiO_2_ chromatography of the HILIC fractions. This demonstrated the feasibility of performing large-scale quantitative phosphoproteomics on submilligram amounts of protein that could be applied to cell material of low abundance.

Although, early studies on peptide separation by HILIC mainly focused on its resolution power and orthogonality as a first fractionation method in a multidimensional set up, there have also been reports evaluating its signal intensities and its applicability in an online HILIC-ES-MS as an alternative to RP-ES-LC. The high organic content used in HILIC results in a peptide signal increase by a factor of 2–10 fold in 88% of cases investigated (81 peptides), compared with RPLC, thus improving the sensitivity of both peptide detection and quantification [42]. Maximum sensitivity was obtained when using amide columns without any salt additives. Yang et al. [43] meanwhile have evaluated the different stationary phases used in HILIC, addressing the effect of mobile phase composition on peak efficiencies with an online HILIC-ES-MS system using peptide mixtures and protein digests. This showed that the use of HILIC-ES-MS provided complementary separation selectivity to RPLC-ES-MS and offered the capability to identify unique peptides, thus highlighting its potential in proteomic applications.

In addition, Horie et al. described the use of a meter-scale monolithic silica capillary column modified with urea functional groups for use in the HILIC mode, which provided highly orthogonal separation to RPLC with sufficient peak capacity, as well as highly sensitive detection for tryptic peptides. In effect, they reported on average ∼5-fold increase in the peak response for commonly identified tryptic peptides due to the high acetonitrile concentration in the HILIC mobile phase suggesting its application as a complementary tool to increase proteome coverage in proteomics studies [44].

In this study, we extend the characterization of HILIC to evaluate its applications in proteomics workflows beyond the enrichment of hydrophilic analytes. Specifically, we systematically evaluate the performance of HILIC against the popular, hydrophilic strong anion exchange (hSAX) method of peptide fractionation, which separates peptides based on their charge [45].

## 2 Materials and methods

### 2.1 Cell culture

U2OS osteosarcoma cancer cells were obtained from the European Collection of Cell Cultures and grown in Dulbecco's Modified Eagle Medium (Lonza) supplemented with 10% fetal bovine serum (Gibco), 50 units/mL penicillin (Lonza) and 50 μg/mL streptomycin (Lonza) for no more than 30 passages at 37 °C and 5% CO_2_.

### 2.2 Protein extraction and proteolytic digestion in solution

For protein extraction, cells were washed twice with cold PBS and then lysed in 0.3–1.0 mL urea lysis buffer (8 M urea, 100 mM triethyl ammonium bicarbonate (TEAB) pH 8.5, Roche protease inhibitors, Roche PhosStop). Lysates were sonicated on ice (6 cycles, 30% power, 30 s). Proteins were reduced with TCEP (25 mM), for 15 min at room temperature and alkylated with iodoacetamide (50 mM), in the dark for 45 min at room temperature. Lysates were diluted with digest buffer (100 mM TEAB) to a final concentration of 4 M urea and digested overnight at 37 °C with endoprotease Lys-C (Wako Chemicals, Japan) using an enzyme to substrate ratio of 1:50. The digest was diluted further using 100 mM TEAB to a final concentration of 0.8 M urea and subjected to a second digestion using trypsin (Promega) in a 1:50 ratio. Finally, the digestion was quenched by adding trifluoroacetic acid (TFA) to a final concentration of 1% (v:v).

### 2.3 Peptide desalting and solid phase extraction

Prior to fractionation, the peptide samples were desalted using C18 Sep-Pak cartridges (Waters). Cartridges were first activated with Acetonitrile (ACN) and equilibrated with 50% ACN in water according to the manufacturer's protocol. The sample was loaded and washed 4 times with 500 μL water containing 0.1% TFA. The peptides were eluted into a fresh Eppendorf tube with 800 μL 50% ACN containing. The peptides are then dried in vacuo.

### 2.4 Off-line HILIC fractionation

HILIC was performed on a Dionex UltiMate 3000 (Thermo Scientific) using a similar protocol to the method described previously [6], [18], [46].

The dried peptides were redissolved in 80% ACN incorporating 0.1% TFA. The peptides were resolved on TSK-gel amide 80-column (TOSOH) using an inverted organic gradient of solvent A (water, 0.1% TFA) and solvent B (ACN, 0.1% TFA). The fractions were collected in deep well 96 well plate. They were dried and redissolved in 5% formic acid (FA).

### 2.5 Off-line hSAX

hSAX was performed on a Dionex UltiMate 3000 (Thermo Scientific) using a similar protocol to the hSAX method described previously[6], [46].

Briefly, tryptic peptides were desalted using Sep-Pak-C18 SPE cartridges (Waters), dried, and dissolved in 50 mM borate, pH 9.3. They were then loaded on AS24 strong anion exchange column and fractionated using an exponential elution gradient from 100% solvent A (10 mM sodium borate, pH 9.3) to 100% solvent B (10 mM sodium borate, pH 9.3 + 0.5 M sodium chloride) using a flow rate of 250 μL min^−1^.

Fractions were collected into a 96-well plate from 5 to 55 min to give 16 fractions. They were acidified and desalted using Sep-Pak-C18 solid-phase extraction (SPE) plates (Waters). The plates were first wetted with 50% acetonitrile (ACN) in water, washed and equilibrated with water containing 0.1% TFA. The acidified peptide fractions were loaded onto the plates, washed with water containing 0.1% FA and then eluted with 300 μL 50% aqueous ACN containing 0.1% TFA. The desalted hSAX fractions were dried in vacuo and redissolved in 5% FA prior to RP-LC–MS.

The elution programme was 100% buffer A for 10 min, continued by a short (1 min) gradient of 0–3% of buffer B, followed by a gradient of 3–15% for 19 min, a 15–45% gradient for 15 min and a 45–100% gradient for 2 min. At the end of the gradient the column was kept at 100% buffer B for 7 min and then for 10 min in buffer A.

### 2.6 Online RP-LC–MS analysis

The peptide samples were dissolved in 5% FA. Their concentration was determined using CBQCA assay (Life Technologies).

RP-LC was performed using a Dionex RSLC nano HPLC (Thermo Scientific). Peptides (1 μg) were injected onto a 0.3 mm id × 5 mm PepMap-C18 pre-column and chromatographed on a 75 μm × 15 cm PepMap-C18. Using the following mobile phases: 2% ACN incorporating 0.1% FA (solvent A) and 80% ACN incorporating 0.1% FA (solvent B), peptides were resolved using a linear gradient from 5% B to 35% B over 156 min with a constant flow of 200 nL min^−1^. The peptide eluent flowed into a nano-electrospray emitter at the sampling region of a Q-Exactive Orbitrap mass spectrometer (Thermo Scientific). The electrospray process was initiated by applying a 2.5 kV to liquid junction of the emitter and the data were acquired under the control of Xcalibur (Thermo Scientific) in data dependent mode. The MS survey scan (MS1) was performed using a resolution of 60,000. The dependent HCD-MS2 events were performed at a resolution of 17,500. Precursor ion charge state screening was enabled allowing the rejection of singly charged ions as well as ions with all unassigned charge states.

### 2.7 Data analysis

The raw MS data from the Q-Exactive Orbitrap (Thermo Scientific) were processed with the MaxQuant software package (version 1.3.0.5). Proteins and peptides were identified against the UniProt reference proteome database (August 2013) using the Andromeda search engine [47], [48]. The following search parameters were used: mass deviation of 6 ppm on the precursor and 0.5 Da on the fragment ions; Tryp/P for enzyme specificity; two missed cleavages. Carbamidomethylation on cysteine was set as a fixed modification. Oxidation on methionine; phosphorylation on serine, threonine, and tyrosine; hydroxylation on proline and acetylation at the protein N-terminus were set as variable modifications. Thresholds for the identification of phosphopeptides were Delta Score = 6 and Andromeda score = 40. The false discovery rate was set to 5% for positive identification of proteins, peptides, and phosphorylation sites.

Most of the subsequent data analysis was done in R version 3.1.3 [49] using Rstudio 0.98.1091 and the package ggplot2 [50]; the sequence coverage analysis was done using Perseus 1.5.1.6 [47] and the GO-terms enrichment analysis using the Cytoscape [51], [52] app BiNGO.

## 3 Results and discussion

**Fig. 1:**
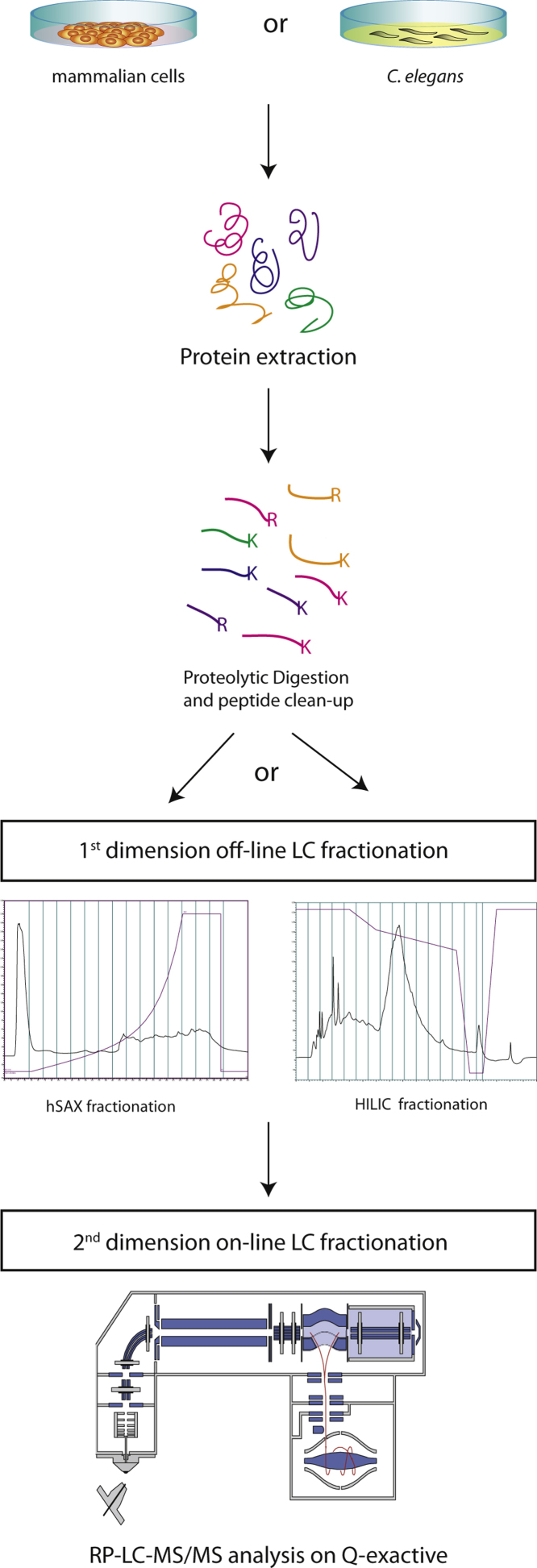
Schematic representation of the proteomics workflow. Protein samples were purified from cultured human cells, or nematodes, and digested with trypsin and Lys-C into individual peptides. To reduce sample complexity, peptides were subjected to “off line” sub-fractionation, either HILIC or hSAX, prior to analysis by LC–MS.

In this study we compared two 2D-LC setups, i.e., HILIC–RP-LC/MS and hSAX–RP-LC/MS, using unfractionated cell lysates from both cultured mammalian cells and from nematodes (Fig. 1). To facilitate a meaningful comparison of these two approaches, we took into consideration the differences in scale and practical implementation of both techniques, including sensitivity levels, system volumes/flow rates and fraction collection. We note, for example, that it is not possible to use the same amount of starting material for each method without either diluting the sample, or overloading one or other of the systems.

Peptide fractionation using hSAX provides good separation when loading relatively low (e.g. ∼100 μg) amounts of material. However, this in our experience is below the maximum practical loading capacity of hSAX, allowing us to increase the amount of peptides injected to limit sample dilution. In contrast, HILIC has a maximum loading capacity for peptides in the order of milligrams, while a minimum of ∼500 μg is required to achieve reasonable separation. Given this intrinsic difference in loading capacities for hSAX and HILIC, in the following experiments to allow us to load equal amounts of material on both set-ups, we chose a concentration of 500 μg that was near the lower limit for HILIC separation to avoid overloading the capacity of the hSAX system.

To ensure robustness and stability of the RP-LC–MS analysis we have also injected 1 μg of each fraction on the RP-C18 column, determined using a fluorescent assay (see Section 2 for details) and used the same standard RP-LC–MS method in each case (summarized in Fig. 1).

### 3.1 Comparing resolution of HILIC and hSAX

**Fig. 2:**
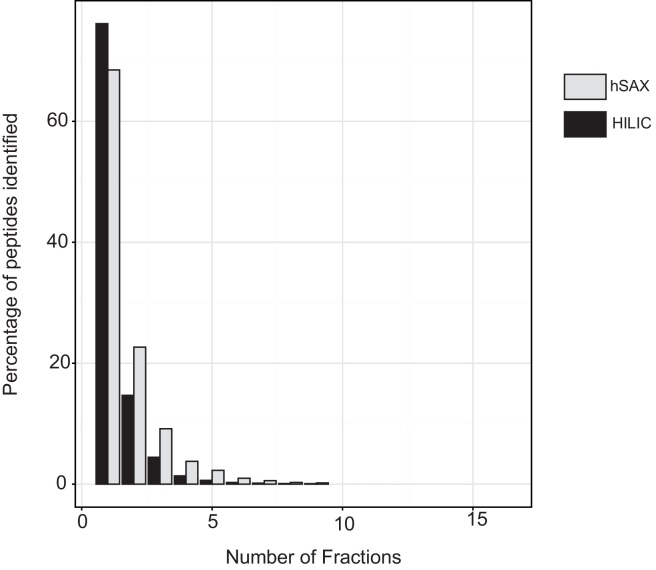
Resolution of hSAX and HILIC separation. Each bar represents the percentage of all peptide evidences from the combined dataset spanning the corresponding number of fractions in the HILIC (black) or hSAX (grey) datasets.

To assess the resolution of chromatographic separation by HILIC and hSAX we have taken the approach of measuring the number of peptides that are only identified in a single fraction and measuring the degree of overlap between adjacent fractions. Higher resolution is obtained when a given peptide is only present in one fraction (or in small number of fractions). Thus, for both HILIC and hSAX we compared the number of peptides identified in a single fraction, two fractions and so on and the results are summarized in Fig. 2. This shows a slightly superior resolution for HILIC where >70% of peptides were observed in a single fraction.

### 3.2 Comparing orthogonal behaviour of HILIC and hSAX with RP-LC

**Fig. 3:**
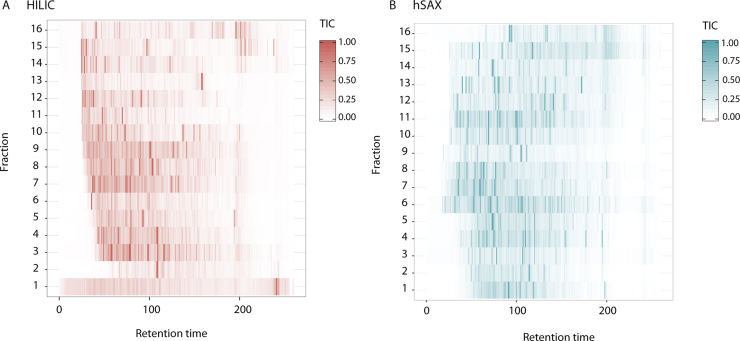
Comparison of the orthogonality of HILIC-RP and hSAX-RP. The figure shows a heatmap of total ion current (TIC), scaled to respective highest value, for each fraction from a (A) HILIC and (B) hSAX method, across retention time.

Next, we compared to what extent the separation properties of HILIC and hSAX were orthogonal with RP-LC (Fig. 3). Both methods show good orthogonality with RP-LC as can be seen from the distribution of peptide intensities across the RP-LC chromatogram for HILIC fractionated peptides (Fig. 3A) and hSAX fractionated peptides (Fig. 3B). From this figure, we can see that there is a broad distribution of ions across the retention time resulting in a wide separation of peptides across the 2D-space.

In addition, we note that while both methods offer good orthogonality they are not identical. This suggests that each technique may have intrinsic specificities that would be relevant to their use in proteomics workflows.

### 3.3 Exploring the consequences of orthogonality

#### 3.3.1 Depth of proteome coverage

**Fig. 4:**
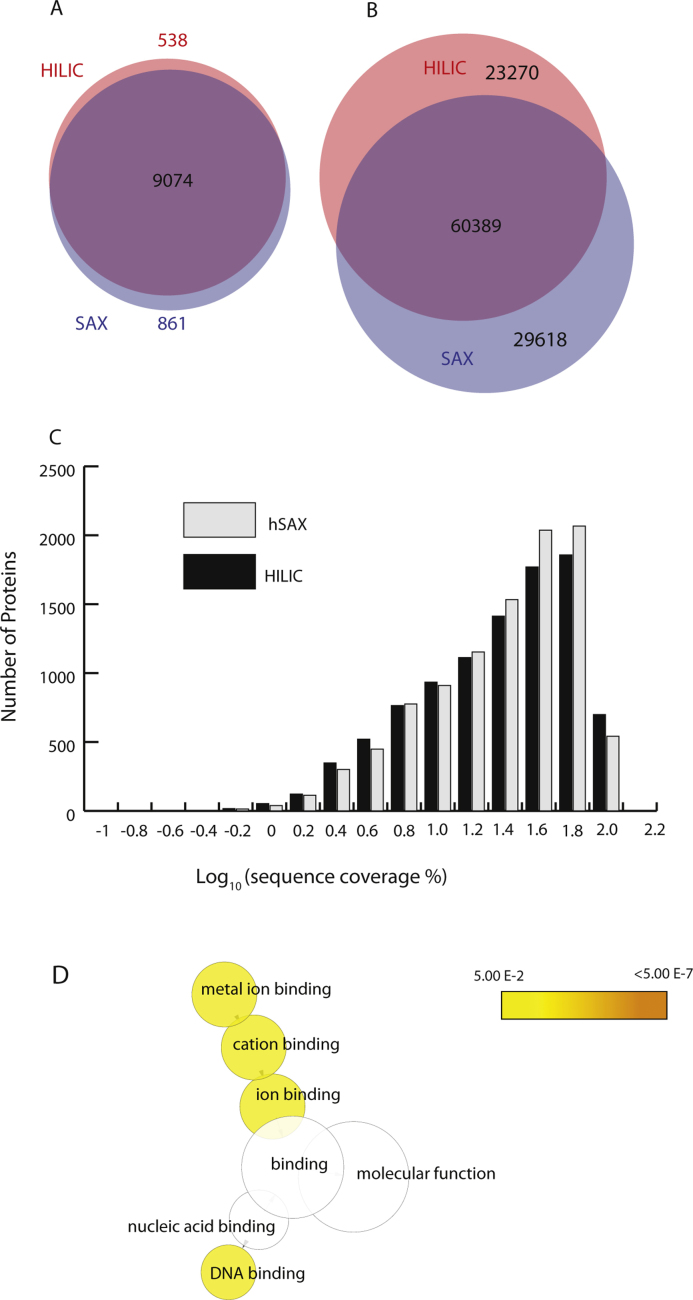
Comparison of the performance of HILIC and hSAX. Euler diagrams of (A) proteins and (B) peptides identified in HILIC (red) and hSAX (blue) datasets. (C) Sequence coverage of proteins identified using HILIC and hSAX. (D) Molecular function GO-terms enriched in the hSAX dataset. No specific GO term was found to be enriched in proteins exclusive to the HILIC dataset. (For interpretation of the references to colour in this figure legend, the reader is referred to the web version of the article.)

Not surprisingly, incorporating either the HILIC, or hSAX methods into the MS workflow allowed a substantial increase in the depth of the proteome measured, in comparison with using RP-LC alone. However, as expected, based on the different physico-chemical properties used to fractionate the peptides, HILIC and hSAX favour different subsets of peptides and proteins. For example, when analyzing extracts of U2OS cells, both set-ups allowed the identification of >9,500 proteins, with hSAX identifying 9,935 proteins and HILIC identifying 9,612 proteins, (Fig. 4A). We note that even though here hSAX identifies slightly (∼3%) more proteins, there is still a subset of specific proteins (∼500) that are only identified in the HILIC–RP-LC experiment. Interestingly, this HILIC-specific group mainly corresponds to proteins that are identified by post-translationally modified peptides (also referred to as site modifications). As discussed further below, this highlights a specific advantage of using HILIC when the identification of PTM-modified proteins is highly relevant to the biological experiment involved.

When looking at the total number of peptides identified, as opposed to proteins, hSAX outperforms HILIC, here identifying more total peptides (Fig. 4B). However, even though hSAX identified substantially more peptides than HILIC, there is still a subset of peptides that were exclusively identified by HILIC, corresponding predominantly to hydrophilic and/or heavily modified peptides. Despite the higher overall number of peptides identified in the hSAX–RP-LC setup, it is significant that this does not result in a dramatic increase in the average protein sequence coverage from that measured by HILIC-RP. Instead we observe that the two 2D-LC techniques are on par with each other, with ∼27% average sequence coverage for proteins identified by either hSAX–RP-LC, or HILIC–RP-LC (Fig. 4C).

It should be noted that in this study we have specifically analyzed peptides resulting from the double digestion of the proteome with trypsin + Lys-C, i.e., essentially tryptic peptides. These peptides have the advantage of possessing a basic residue at their C-terminus, which facilitates ionization under the conditions of online RP-LC and aids efficient fragmentation using collision induced dissociation (CID). However, amongst the set of tryptic peptides generated, a significant proportion (∼56%) are too short (<6 amino acid residues) to be identified reliably by LC–MS/MS based methods [53].

One approach to increase the average protein sequence coverage further could be to employ parallel digestions using several proteases with different cleavage specificities, subsequently combining the results. This approach was reported recently to result in a significant increase in sequence coverage, which is further improved by using different activation methods during the tandem MS experiment [53], [54]. The multiple protease approach will result in peptides that are heterogeneous with regards to the position of basic residues and will not therefore be ideal for CID. For example, peptides that contain internal basic residues will give rise to internal fragments that are usually unassigned by current database search algorithms and their identification would benefit from using alternative activation techniques, such as electron transfer dissociation (ETD) and more recently ultraviolet photodissociation (UVPD).

#### 3.3.2 Gene ontology analysis

As demonstrated above, in addition to a major overlap, hSAX and HILIC favour detection of different subsets of peptides and proteins. To investigate the natures of the differences in protein identifications, we employed Gene Ontology analysis. To do this, the protein lists were submitted for statistical testing to identify the functional categories of enriched genes defined by Gene Ontology (GO) using the BiNGO app from Cytoscape [51], [52] as well as using DAVID (http://david.abcc.ncifcrf.gov/home.jsp) [52], [55]. The results reveal that hSAX clearly enriches for specific classes of protein sequence features, especially different types of the zinc finger regions C2H2. Amongst the GO-molecular function terms enriched are ion binding, DNA binding and metal binding. Fig. 4D shows the Cytoscape GO term networks, highlighting biological processes that are significantly enriched in the hSAX protein list. The full results of the GO analysis are shown in Supplementary Table 1.

Supplementary table related to this article can be found, in the online version, at http://dx.doi.org/10.1016/j.ijms.2015.07.029.



Interestingly, a GO term analysis on a similar number of proteins specifically detected in the HILIC fraction showed no significant enrichment of either sequence motifs, or any GO terms associated with function (data not shown). This is consistent with the fact that HILIC uses polarity/hydrophilicity to fractionate peptides, a property that shows little or no specificity for functional classes of proteins. In contrast, hSAX will preferentially enrich classes of proteins that contain highly charged regions, such as nucleic acid binding proteins. We infer that HILIC displays minimal bias relating to GO-terms beyond any intrinsic sampling bias inherent to the extract preparation methods.

### 3.4 Is HILIC undersampling?

**Fig. 5:**
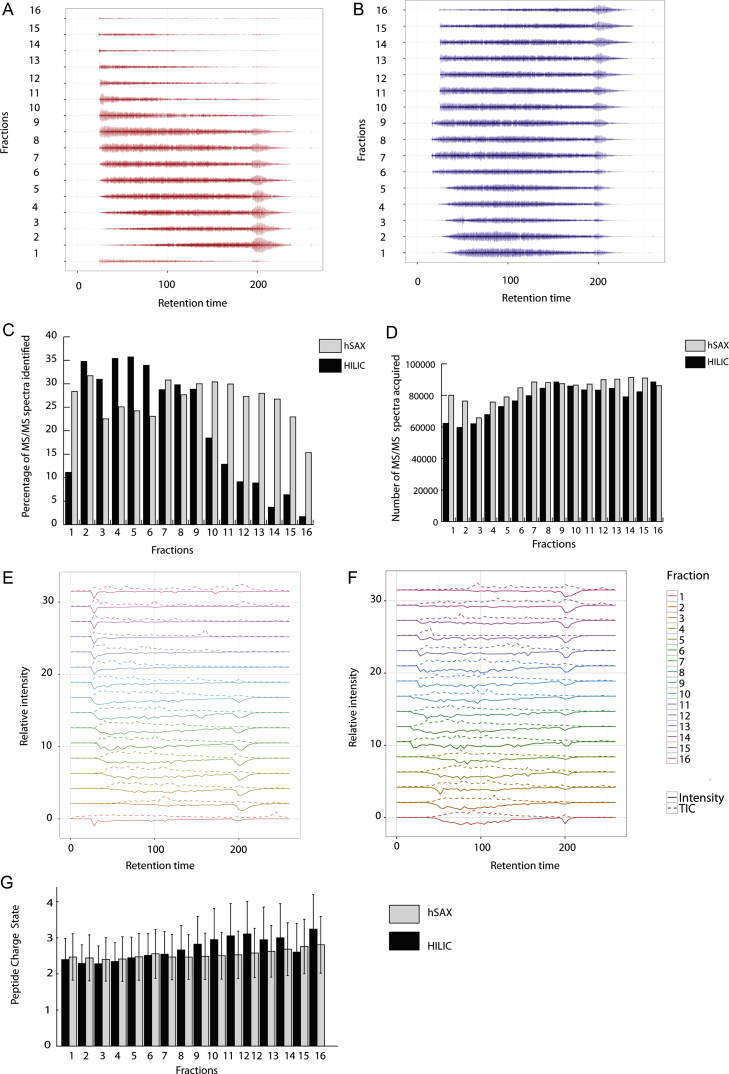
Comparison of the efficiency of the conversion of MS/MS spectra into peptide identifications between HILIC and hSAX. Representation of the sequenced evidences profile of each of the (A) HILIC and (B) SAX fractions across runtime. This plot represents, for each fraction, the retention length of all identified peptide evidences, represented as a segment centred on their retention time, where each segment's colour intensity is a function of evidence intensity. Segment positions are dodged on either side of the horizontal axis to avoid overlap, with the least intense ones towards the periphery; hence, the width of the segment cloud is a function of the number of peptides eluting at the time point considered. (C) Percentage of MS/MS spectra which resulted in a successful identification, i.e., in a peptide-spectrum match (PSM), per fraction. (D) Number of MS/MS spectra acquired per HILIC and hSAX fraction. (E and F) Scaled relative intensity profile for total ion current (TIC, dashed line) and successfully identified spectra (“evidences”, full line, mirrored) for HILIC and hSAX, respectively. (G) Mean peptide charge per HILIC and hSAX fraction.

Next, we investigated possible reasons that could explain the lower numbers of peptides identified by HILIC-RP. We started by examining the number of successful peptide identifications per fraction for each of the 2D-LC set-ups (Fig. 5). This shows a dramatic decrease in the number of peptides identified in the later fractions of HILIC (Fig. 5A). In fact, there is a gradual decrease in the number of peptides identified from fraction 9 to fraction 16, with successful peptide identifications made early in the RP-LC gradient in keeping with the increased hydrophilic character of these peptides. In contrast, the number of peptide identifications is uniformly distributed across the hSAX fractions and across the RP-LC chromatogram (Fig. 5B), suggesting that most/all of the hSAX fractions are similar in terms of their hydrophobicity.

Analysis of the percentage of successful MS2 identifications across fractions in each experiment shows a dramatic decrease in the number of successful MS2 identification in HILIC, as compared with hSAX fractionation (Fig. 5C). A possible explanation is that the later HILIC fractions are largely empty, with very few peptides that can be selected for MS/MS. However, this is not the case, as shown by looking at the numbers of tandem MS spectra acquired across all of the HILIC fractions, which are similar to the number of spectra acquired for hSAX fractions (Fig. 5D). This shows that the total number of MS/MS spectra acquired is relatively constant across the HILIC fractions.

When, for each HILIC and hSAX fraction, the total intensities from the raw chromatogram (total ion current, cf. Fig. 3) and from the successfully sequenced spectra are plotted side by side (Fig. 5E and F for HILIC and hSAX, respectively), it becomes apparent that while the later HILIC fractions do appear to be lower complexity than the earlier ones, they are also yielding relatively fewer sequenced evidences. We conclude therefore that it is the percentage of spectra that led to successful peptide identifications that has dropped in the later fractions of HILIC.

Several factors may be contributing to the observed decrease in successful peptide assignments from the spectra recorded from the later HILIC fractions. First, these later fractions may be preferentially enriched in peptides containing one or more post translational modifications that we have not included in our database searches and as a result we were blind to these peptides. For example, HILIC has been reported to successfully enrich for O-GlcNAc containing peptides and sialic acid containing glycopeptides [56], amongst other sugars, which were not used as variable modifications when interrogating the database in this study [57]. Similarly, these peptides may carry other known modifications that were not searched for and/or rare, or even novel, modifications, which may be unknown to us. Therefore, these peptides could be ideal targets for future analysis by de novo sequencing, rather than seeking to identify them by database matching.

Second, there may be a decline in the quality of the spectra in the later HILIC fractions, reducing the number of spectra that are good enough for successful peptide identification. Successful peptide identification can be achieved only when product ions from a complete or nearly complete distribution of amide backbone cleavages are observed in the corresponding MS/MS spectrum. This could arise if these fractions are enriched in peptides that are modified in a way that alters either their behaviour, or fragmentation pattern, when subjected to HCD (and more generally CID).

Briefly, in this process, peptides that are protonated more or less randomly on backbone amide nitrogen atoms [58] are collided with an inert gas. Imparted kinetic energy is converted to vibrational energy, which is then rapidly distributed throughout all covalent bonds in the peptide (picosecond time scale). Fragment ions are formed when the internal energy of the ion exceeds the activation barrier required for a particular bond cleavage. Fragmentation of protonated amide bonds affords a series of complementary product ions of types b and y [59], [60], [61], which allow assignment of a peptide sequence to a precursor ion.

In this case the peptides in later HILIC fractions may not produce ideal fragmentation under the CID regime and hence not yield assignable MS2 spectra. For example, they may be heavily modified, by carrying several phosphate groups, or other labile groups, that readily dissociate by a lower energy pathway than that involved in the cleavage of the amide linkage, thus reducing the extent of backbone cleavages and so making the spectra difficult to assign. For example, in the gas phase, the phosphate competes with the peptide backbone as a preferred site of protonation and consequently, after collisional activation, undergoes nucleophilic displacement by a neighboring amide carbonyl group. The resulting [(M+*n*H)^*n*+^−H_3_PO_4_] product ions often constitute ≥85% of the fragment ions observed under the low-energy CID conditions.

Identification of such peptides could benefit from using alternative activation methods, such as electron transfer dissociation (ETD) [62], which results in backbone cleavage even in the presence of labile PTMs. This is due to the fact that ETD, like its predecessor ECD, is independent of amide bond protonation and occurs on a shorter time scale compared with internal energy distribution so that heavily modified peptides fragment more or less randomly along the peptide backbone and are easily sequenced.

It is also possible that the unassigned peptides are highly charged, so that when subjected to CID they give rise to MS2 spectra that are too complicated for reliable database searching and identification. The presence of multiple basic residues in the sequence inhibits random protonation along the peptide backbone and thus reduces the extent of backbone cleavage, which is commonly accepted to occur predominantly through charge-directed pathways (the mobile proton model) [63].

Again, exploring other activation methods could be beneficial. For example, Coon and co-workers have reported that highly charged species gave more useful sequence information under ETD while lower charged species (2+ and 3+ charge states) gave more successful assignments under the CID regime leading to the introduction of decision tree based proteomics to improve the sequence coverage of the proteome [64]. We note that in Fig. 5E, the hSAX fractions have a relatively constant distribution of charge states (2+ and 3+) which are consistent with them showing more constant MS2 assignments throughout the fractions in contrast with the unequal distribution of ion charges across the HILIC fractions.

**Fig. 6:**
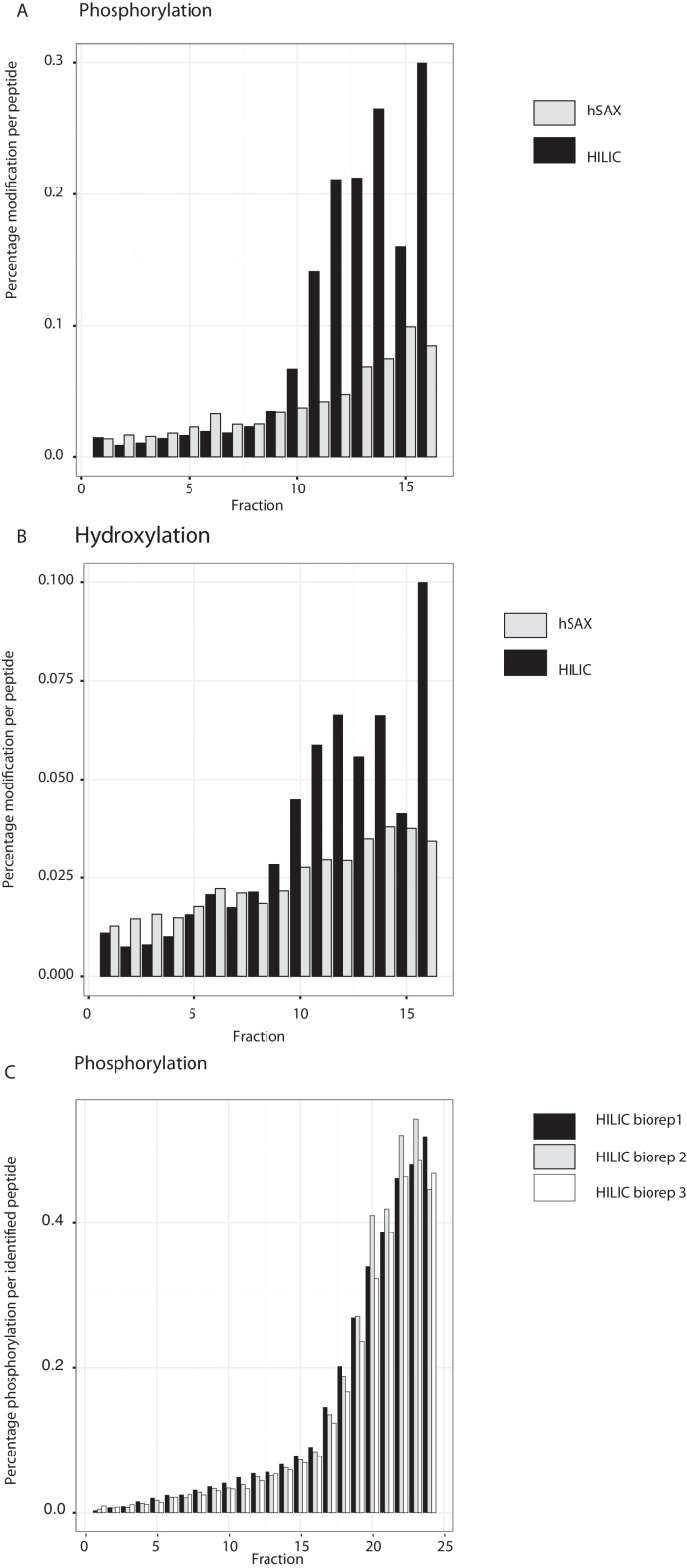
Distribution of examples of hydrophilic PTMs across HILIC and hSAX fractions. Mean number of (A) phosphorylations and (B) hydroxy-proline modifications detected per number of peptides across fractions for HILIC and hSAX. (C) Number of phosphorylations per peptide for three biological replicates fractionated using the same HILIC method.

Consistent with the possibilities discussed above, we indeed observe more phosphorylated peptides in the later HILIC fractions, where we have increased the polarity of the mobile phase and reduced its organic content (cf. Fig. 6). Analysis of extracts prepared from both human cell lines and nematodes shows a consistent trend, with ∼30% of the peptides in the latter fractions having hydrophilic modifications, such as phosphorylation and/or proline hydroxylation (Fig. 6).

In summary, we observe a gradient of peptide identification efficiency across the HILIC fractions that may reflect the preferential enrichment in the later fractions of classes of hydrophilic peptides that are currently difficult to identify efficiently using conventional database search algorithms.

## 4 Conclusion

Given the diversity of physico-chemical properties of proteins and their post-translationally modified forms, it is likely that there is no ‘one size fits all’ fractionation method that will allow perfectly efficient detection and measurement of all proteins and peptides in a single experimental setup. If the goal of a given proteomic study is to obtain the most comprehensive measurement of all forms of proteins in a cell, tissue or organism, then it is likely that more than one analytical technique will be required to maximize coverage. There are available now multiple chromatography setups that can be linked with tandem MS analyses and in this study we have compared specifically the performance of combining conventional RP-LC–MS with either HILIC, or hSAX, respectively. Both methods allowed an increase in the depth of proteome coverage as opposed to using RP-LC alone. In addition, the data show that both methods resulted in approximately equal numbers of protein identifications with similar average sequence coverage; despite the higher number of total peptide identifications obtained using hSAX as opposed to HILIC.

Overall the data in this study show that hSAX is highly orthogonal with RP-LC and can be easily applied in large-scale proteomics, providing deep proteome analysis with good sequence coverage. The data also show that hSAX has slightly lower resolution than HILIC, in keeping with recent reports by Trost and co-workers [45], who have shown ∼55% of peptides eluting in one fraction when using hSAX, as compared with 69% for RP-LC. We also find that hSAX displays some bias towards preferential enrichment of peptides from specific classes of proteins, particularly those with highly charged domains. HILIC provides a robust and reproducible separation method for high throughput proteomics. Like hSAX, it helps to increase the depth of the proteome detected and is particularly useful in enhancing the detection of a subset of proteins that may otherwise be underrepresented, especially including proteins with post translational modifications. This can be particularly useful for biological experiments where it is important to detect the roles of specific hydrophilic PTMs, such as phosphorylation and proline hydroxylation, especially when it is not practical to include PTM-enrichment strategies in the experimental workflow.

It is likely that the performance of HILIC can be improved even further. For example, in this study, we observed undersampling of peptides in the earlier HILIC fractions. By analyzing the hydrophobic portion of the HILIC chromatogram, using standard online RP-LC–MS, no peptides were detected eluting for ∼40 min. As the organic content of the mobile phase increased, peptides of comparable hydrophobicity were then sprayed over a short time period, likely overloading the tandem MS (MS/MS) detection events and thus reducing the overall numbers of peptides detected. A potential way to improve performance would thus be to modify the RP-LC–MS gradient according to the hydrophobicity of the HILIC fractions, hence allowing earlier fractions to be analyzed using a shallower gradient that starts with higher organic content, potentially leading to a greater number of peptide and protein identifications.
